# Correcting misperceptions of gun policy support can foster intergroup cooperation between gun owners and non-gun owners

**DOI:** 10.1371/journal.pone.0268601

**Published:** 2022-06-08

**Authors:** Mark W. Susmann, Graham N. Dixon, Brad J. Bushman, R. Kelly Garrett

**Affiliations:** 1 Department of Computer Science and Engineering, Ohio State University, Columbus, Ohio, United States of America; 2 School of Communication, Ohio State University, Columbus, Ohio, United States of America; Bryant University, UNITED STATES

## Abstract

Past research finds that a majority of gun and non-gun owners support key gun safety policies, yet gun owners tend to underestimate other gun owners’ support for these policies. We predicted that these misperceptions of support might lead gun owners to view non-gun owners as being less similar to themselves, which might undermine intergroup cooperation to promote gun safety policies and fuel intergroup animosity. Importantly, we also predicted that correcting these misperceptions would be an effective way to reduce intergroup division and enhance intergroup cooperation. We tested these predictions across two studies in which participants were randomly assigned to read information designed to correct misperceptions of gun owner support or to read other, control information. Across both studies, we find that correcting gun owners’ misperceptions of gun owner support for gun safety policies leads to greater perceptions of identity overlap between gun and non-gun owners, greater willingness to work with each other to promote gun safety policies, and less negative affect towards each other. This suggests that correcting gun owner misperceptions of gun owners’ support for gun safety policies might be an effective intervention to facilitate intergroup cooperation to promote these policies. Therefore, efforts to promote gun safety policies might benefit from educating gun owners about the degree of support for these policies that already exists among gun owners. Doing so might present a simple and cost-effective way to mobilize gun owners in support of these policies.

## Introduction

Mark Twain once said: “The trouble with the world is not that people know too little; it’s that they know so many things that just aren’t so.” Effective action to combat social problems is often not aided by inaccurate beliefs surrounding those problems. The United States currently faces such a challenge pertaining to gun violence. Gun violence is currently costing the lives of thousands of Americans every year, with approximately 45,000 Americans killed by gun violence in 2020 alone [1]. Federal legislative action to reduce gun violence has been limited despite widespread public support among both gun and non-gun owners for gun safety policies such as universal background checks [2] and federal mandatory waiting periods [3]. Though the question of whether gun safety policies reduce the incidence of gun violence remains the subject of much debate [4, 5], there is reason to believe, particularly when examining gun violence from a public health perspective, that the implementation of gun safety measures could have a meaningful impact on reducing gun violence [6, 7].

One possible reason public support for gun safety policies has not been more influential is that Americans regularly misjudge how controversial these policies are. Both gun and non-gun owners widely underestimate the percentage of gun owners who support the policies [8]. This misperception reduces gun owners’ willingness to voice their opinions on gun safety policies publicly [9], which contributes to a vicious cycle by exacerbating the misperception that gun owners systematically oppose such policies.

Research on social norms suggests that misperceptions about gun owners’ support for gun safety policies could prevent these supporters from acting on their attitudes. Individuals are often more influenced by attitudes that they perceive as normative to their ingroup than by those that are normative to the outgroup [10]. The prospect of acting against a perceived ingroup norm could also elicit fears of being ostracized from the group in retaliation [11]. These dynamics could further disincentivize gun owners from promoting gun safety policies themselves and make them less willing to work with non-gun owners to promote such policies [12, 13].

Among gun owners inclined to support gun safety policies, misperceptions of gun owner support could also hinder their cooperation with non-gun owners by promoting intergroup division. Gun ownership plays a central role in many people’s recreational lives, and it has important connections to notions of self-defense [14]. Additionally, the politicization of gun regulation, combined with high levels of political affective polarization in the United States [15], suggest that gun ownership status has become an influential social identity for many Americans. Indeed, gun owners tend to show distinct political behaviors from non-gun owners (such as systematically voting for different political candidates) in a way that is similar to how other meaningful political social identities operate [16], and it has been argued that political interest groups, such as the National Rifle Association, have purposefully cultivated a distinct gun owner social identity to help mobilize gun owners against gun safety policies [17]. Those who share their gun ownership status are ingroup members and those who do not belong to the outgroup. Tendencies to make such group distinctions could be consequential. People tend to be preferential to ingroup member needs over outgroup member needs [18], even when group membership has been arbitrarily assigned [19]. Likewise, people often overestimate differences in opinions between ingroup and outgroup members [20]. In the context of gun ownership, this tendency could be exacerbated further by misperceptions about gun owner support for gun safety policies, which could contribute to both groups perceiving more intergroup division than exists. Exaggerated perceptions of intergroup dissimilarity could heighten ingroup preferential tendencies and undermine willingness to cooperate with outgroup members to promote gun safety policies [21, 22]. Additionally, these processes could heighten intergroup animosity [23].

All of this serves to make policy change less likely because policy makers who align themselves with gun-owner interests are unlikely to be persuaded by advocacy dominated by non-gun owners [13]. Thus, enhancing gun owners’ willingness to cooperate with non-gun owners to advance gun safety laws could be a uniquely effective tool for promoting political action on this important social issue.

Providing evidence that gun and non-gun owners similarly support gun safety policies could allow gun owners to update their beliefs about normative ingroup attitudes to be more accurate. This could lead to reduced perceptions of intergroup difference and facilitate cooperation with outgroup members to promote gun safety policies. It is possible that providing information to correct misperception about gun owner support would be effective with little sensitivity to how that corrective information is presented. However, the benefits of this approach could also potentially could be constrained in some situations, specifically if the corrective information does not address reasons why misperceptions of gun owner support exist in the first place. Past research examining misinformation correction finds that individuals are more likely to accept correcting information when it provides a clear explanation of the cause of the misinformation [24]. Therefore, explaining the cause of these misperceptions and why they are inaccurate might be more effective at correcting the misperceptions than only stating that the misperceptions are inaccurate.

### Present research

The present research examined the impacts of policy support corrective information on intergroup cooperation surrounding gun safety policies. This research focused on gun owners because we anticipated that the effects of correcting misperceptions would be especially pronounced among this group. Groups purported to represent the interests of American gun owners have been instrumental in blocking adoption of gun safety policies in the United States [25]. To the extent that advocacy from gun owners would be more likely to sway the positions of aligned groups and politicians [13], efforts to mobilize gun owners in support of gun safety policies are expected to be particularly impactful.

We tested four primary hypotheses:

H1: Correcting misperceptions about gun owner support for universal background checks and mandatory waiting periods will lead gun owners to perceive greater group identity overlap between themselves and non-gun owners.

H2: Correcting these misperceptions will lead gun owners to report greater willingness to work with non-gun owners to promote universal background checks and mandatory waiting periods.

H3: Correcting these misperceptions will reduce the amount of negative affect gun owners feel towards non-gun owners.

H4: The effects described in Hypotheses 1–3 will be stronger when the corrective information contains a clear explanation for why the misperception exists than when it does not.

We report two experiments examining these predictions. Experiment 1 (preregistered here: https://osf.io/qw65y/?view_only=a42ce410dc424673a98a031b7c512a29) addressed Hypotheses 1–3, and Experiment 2 (preregistered here: https://osf.io/aemhc/?view_only=2a0ad0157848472789414ec9a382a72a) examined all four hypotheses.

## Experimental methods

### Ethics statement

Ethics approval for these studies was granted by The Ohio State University’s Institutional Review Board (approval number: 2020B0249). Informed consent was given by participants digitally online.

### Participants

Sample sizes were collected in accordance with our preregistered sample size targets with the goal of having approximately 125 participants per cell in each study’s design. In exchange for monetary compensation, 241 gun owners participated in Experiment 1 and 752 in Experiment 2 via Amazon Mechanical Turk. Forty-four participants were excluded from Experiment 1 and 56 were excluded from Study 2 because they were flagged by Qualtrics as likely bots and/or duplicate responses. Additionally, because gun ownership is not evenly distributed between men and women in the United States (39% of men own firearms compared to 22% of women [26]), we statistically controlled for participant gender in all of our analyses to ensure that this demographic difference is not responsible for our observed effects. Two participants did not indicate their gender, so they were also excluded from the analyses. These exclusion rates were higher than hoped, but they are not uncommon among MTurk-based studies [27]. This resulted in valid data for 195 participants (49.7% female), 20 to 79 years old (*M* = 44.94, *SD* = 13.51) in Experiment 1 and 696 participants in Study 2 (41.5% male, 58.0% female, 0.3% non-binary, 0.1% preferred not to say), 19 to 83 years old (*M* = 43.07, *SD* = 13.48). Data for Experiment 1 were collected between April 24^th^ and April 26^th^, 2021, and data for Experiment 2 were collected between June 17^th^ and June 27^th^, 2021.

Manipulations and measures

#### Corrective information manipulations

In Experiment 1, participants were randomly assigned to either a corrective information condition or a no corrective information condition. Those in the corrective information condition were told majorities of gun owners and non-gun owners support universal background checks and mandatory waiting periods and were shown supporting polling data with the percentages of both groups who support the policies. Those in the no corrective information condition were only told a majority of non-gun owners support universal background checks and mandatory waiting periods; no information about gun owner support was provided (see the online supplement for exact experiment materials).

In Experiment 2, participants were randomly assigned to the two conditions used in Study 1 or a third condition that included both corrective and explanatory information. This third condition was the same as the corrective information condition except it included a paragraph explaining why people might hold misperceptions about gun owner support for universal background checks and mandatory waiting periods. Participants were told that people over-estimate how controversial these policies are because a vocal minority of dissenting gun owners tend to dominate discussions about them. This rationale was supported by previous research [28], and data from Experiment 1 showing that individuals who identified more strongly with being a gun owner were more likely to hold negative attitudes towards universal background checks, *b* = -0.37, *se* = .10, *t*(192) = -3.72, *p* < .001, 95% CI [-0.568, -0.175], *r* = .26, and mandatory waiting periods, *b* = -0.50, *se* = .12, *t*(192) = -4.33, *p* < .001, 95% CI [-0.726, -0.272], *r* = .30, and were more likely to share those views with others *b* = 0.61, *se* = .09, *t*(192) = 6.69, *p* < .001, 95% CI [0.428, 0.786], *r* = .43; see the online supplemental materials for details on these measures). However, because the corrective and corrective plus explanatory information conditions did not significantly differ in any of the Experiment 2 analyses, these two conditions were combined for simplicity in the reported analyses (see the online supplement for analyses that separately compare these conditions).

#### Corrective information manipulation check

In both experiments, participants were asked to provide numerical estimates of the percentages of gun owners who support the two gun safety policies using open-ended items. This allowed for assessment of whether the corrective information manipulation had the desired effect on participants perceptions of gun owner support for the policies. Identical questions were asked about non-gun owners’ support for the policies for comparison.

#### Perceived overlap between gun and non-gun owners group identity

Perceptions of identity overlap between gun owners and non-gun owners were assessed in both experiments using a single item adapted from similar measures [29]. Participants were shown a visual scale in which gun owners and non-gun owners were represented as two distinct circles that increasingly overlapped at each level of a seven-point scale (see the supplemental materials for more details and an image of what this measure looked like). Participants were asked to select the number corresponding to the image that best represented the degree to which they thought the groups differ or do not differ from one another.

#### Willingness to work with gun and non-gun owners to promote gun safety policies

Three items assessed participants’ willingness to work with gun owners to promote universal background checks and three assessed willingness to work with non-gun owners. The wording was the same except for the name of the issue being assessed: universal background checks or mandatory waiting periods. Items asked about willingness to work with gun/non-gun owners to advocate for the policies, protest with them in support of the policies, and support organizations led by gun/non-gun owners that advocate for the policies (see the online supplement for details about all measures included in the experiments). Responses to items regarding gun owners and universal background checks were averaged to create a single index (Experiment 1: α = .89; Experiment 2: α = .86), as were the items regarding gun owners and mandatory waiting periods (Experiment 1: α = .88; Experiment 2: α = .88), non-gun owners and universal background checks (Experiment 1: α = .90; Experiment 2: α = .89), and non-gun owners and mandatory waiting periods (Experiment 1: α = .91; Experiment 2: α = .90).

#### Negative affect towards gun and non-gun owners

In both experiments, measures of the negative affect participants towards members of their group (gun owners) and the outgroup (non-gun owners) were adapted from measures of political affective polarization [30, 31]. Participants were asked the extent to which they agreed non-gun owners were describable via several positive and negative traits. Ratings were given on sliding scales from -50 (*Strongly Disagree*) to 50 (*Strongly Agree*). Responses were recoded to a 0–100 scale and responses to positive trait items were reverse-coded and averaged with responses to the negative trait items. This resulted in a single index of negative affect towards non-gun owners, with higher values of the index indicating greater negative affect. Reliability of this index was good (Experiment 1: α = .91; Experiment 2: α = .90). Participants gave the same ratings about gun owners and an index was created in the same way (Experiment 1: α = .91; Experiment 2: α = .91).

### Procedures

After providing written online informed consent, participants in both experiments were asked whether they owned one or more firearms and could only continue if they responded “yes.” Participants then responded to several items unrelated to the focal predictions (see the online supplement for details these measures). In Experiment 1, participants were then randomly assigned to either the no corrective or corrective information condition. Next, they answered the manipulation check and group identity overlap measures followed by additional measures including the willingness to work with gun and non-gun owners to promote gun safety policies and the negative affect measures. Finally, participants completed demographic and screening questions, were debriefed, and thanked for their participation.

In Experiment 2, the procedure was identical except participants were randomly assigned to either the no corrective information, corrective information, or corrective plus explanatory conditions. Study 2 also contained a manipulation of intergroup trust. However, because this manipulation did not significantly impact any of the reported outcomes or moderate the impacts of any of the other effects, we will not discuss it further (see the online supplement for details about this manipulation).

## Results

### Corrective information manipulation check

The corrective information manipulations were successful in both studies. Those in the corrective condition(s) estimated that significantly more gun owners support universal background checks (Experiment 1: *M* = 76.12%, *SD* = 18.09; Experiment 2: *M* = 77.80%, *SD* = 15.65) than those in the no corrective information condition (Experiment 1: *M* = 56.74%, *SD* = 25.85, *b* = 9.67, *se* = 1.58, *t*(192) = 6.12, *p* < .001, 95% CI [6.556, 12.787], *r* = .40; Experiment 2: *M* = 58.64%, *SD* = 25.00, *b* = 9.55, *se* = 0.78, *t*(691) = 12.32, *p* < .001, 95% CI [8.028, 11.074], *r* = .42). Two participants in Experiment 2 did not provide estimates and were excluded from this analysis and one participant provided an impossible estimate (800%), so we recoded that response to 100. The mean difference was significant with or without this recoding.

Participants in the corrective information condition(s) also estimated that significantly more gun owners support mandatory waiting periods (Experiment 1: *M* = 70.19%, *SD* = 18.04; Experiment 2: *M* = 72.04%, *SD* = 16.73) than those in the no corrective information condition (Experiment 1: *M* = 49.36%, *SD* = 26.41, *b* = 10.38, *se* = 1.61, *t*(191) = 6.45, *p* < .001, 95% CI [7.207, 13.558], *r* = .42; Experiment 2: *M* = 48.52%, *SD* = 25.68; *b* = 11.70, *se* = 0.81, *t*(690) = 14.44, *p* < .001, 95% CI [10.111, 13.292], *r* = .48). Note that one participant in Experiment 1 and three participants in Experiment 2 did not complete this measure and were excluded from these analyses. No significant differences emerged between conditions for estimates of non-gun owner support (see the online supplement for these analyses).

### Perceived overlap between gun and non-gun owners group identity

**Fig 1:**
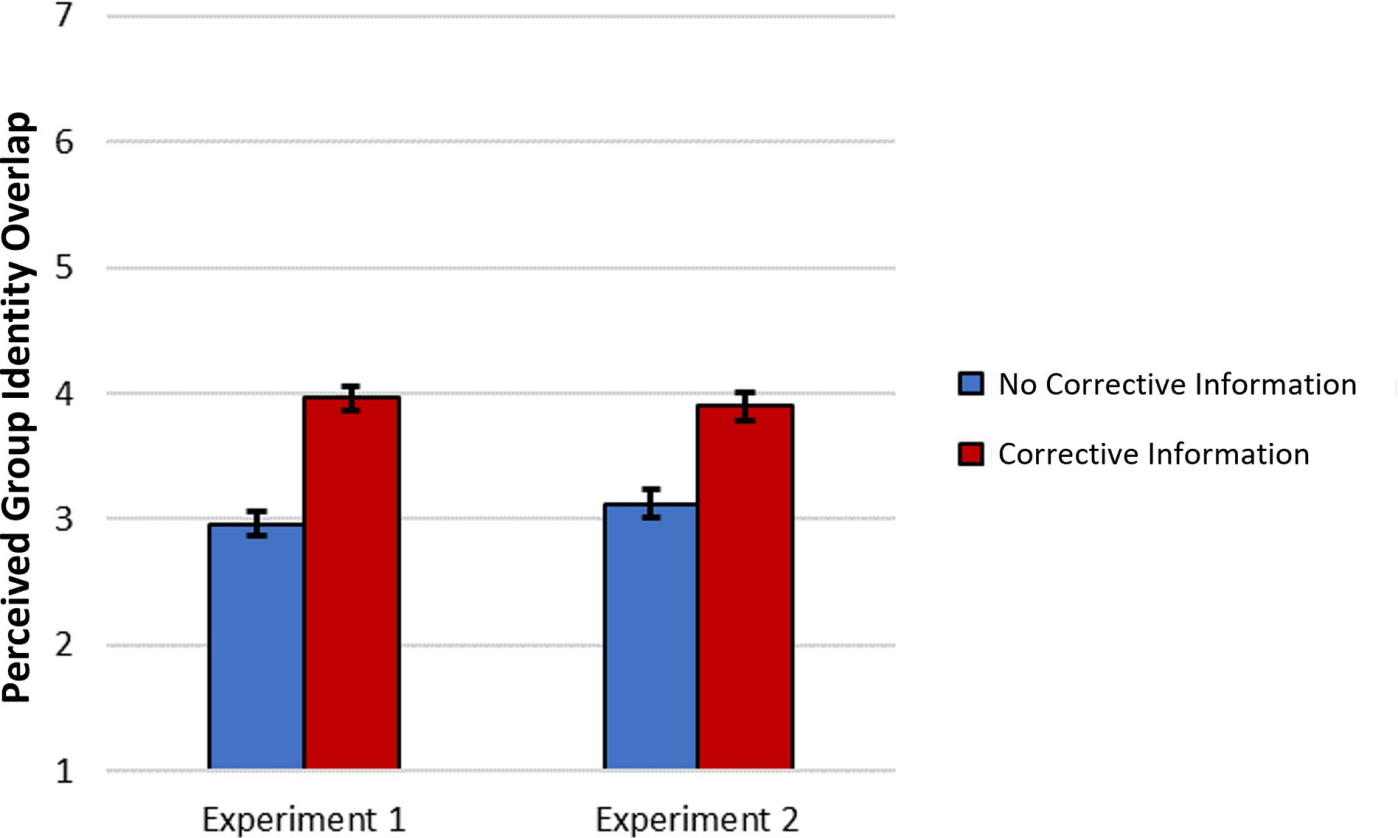
Perceived group identity overlap between gun owners and non-gun owners as a function of corrective information condition data from Experiments 1 (*N* = 195 gun owners) and 2 (*N* = 696 gun owners). Error bars represent 95% confidence intervals.

As predicted, those in the corrective information condition(s) perceived greater identity overlap between gun and non-gun owners (Experiment 1: *M* = 3.96, *SD* = 1.42; Experiment 2: *M* = 3.90, *SD* = 1.52) than those in the no corrective information condition, (Experiment 1: *M* = 2.96, *SD* = 1.33, *b* = 0.50, *se* = 0.10, *t*(192) = 5.06, *p* < .001, 95% CI [0.305, 0.695], *r* = .34; Experiment 2: *M* = 3.12, *SD* = 1.35, *b* = 0.39, *se* = 0.06, *t*(693) = 6.71, *p* < .001, 95% CI [0.277, 0.506], *r* = .25; see Fig 1).

### Willingness to work with gun and non-gun owners to promote gun safety laws

**Fig 2:**
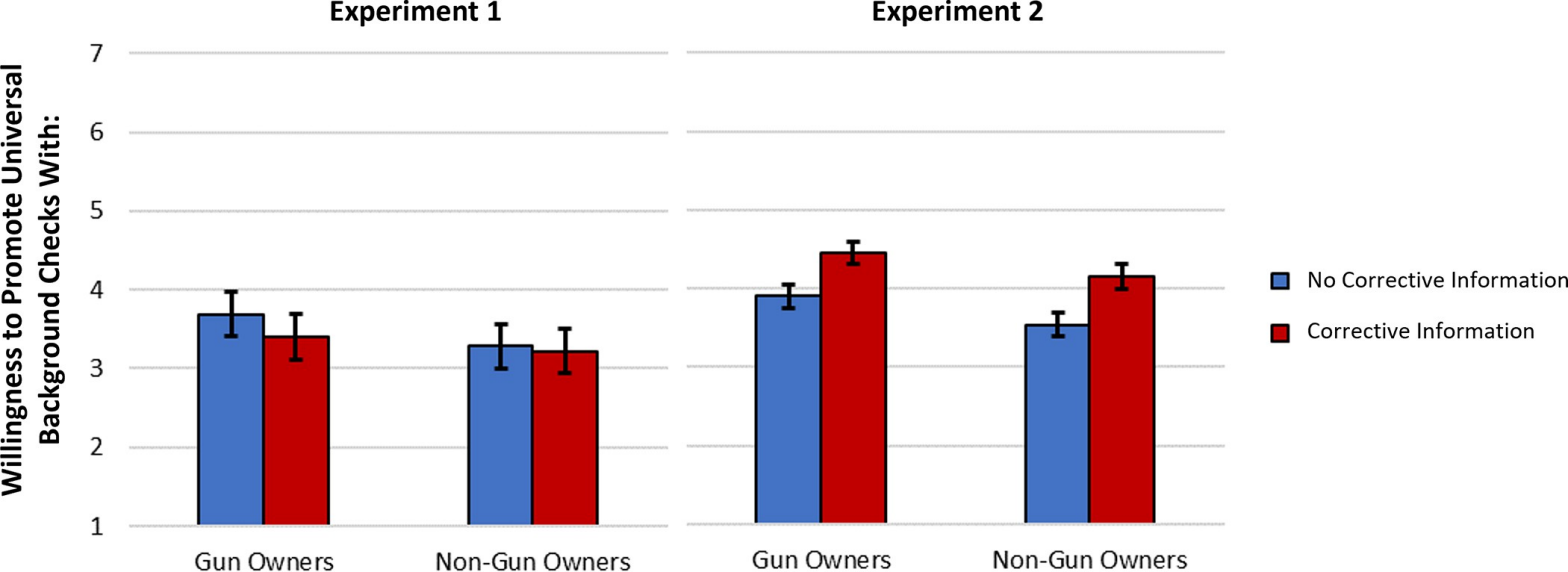
Willingness to promote universal background checks with gun and non-gun owners as a function of group and corrective information condition. Data from Experiments 1 (*N* = 195 gun owners) and 2 (*N* = 696 gun owners). Error bars represent 95% confidence intervals.

Two repeated measures ANOVAs were conducted, one predicting willingness to work with either gun owners or non-gun owners on universal background checks and the other predicting willingness to collaborate with one of these groups on promoting mandatory waiting periods. Outcomes were predicted from the presence or absence of corrective information, which group was being assessed (the “target”, i.e., gun or non-gun owners), and the interaction between these variables while also controlling for participant gender. In both studies, there was a main effect of the group being assessed on universal background checks (Experiment 1: *F*[1, 192] = 10.81, *p* = .001, *η*_*p*_^*2*^ = .05; Experiment 2: *F*[1, 693] = 52.75, *p* < .001, *η*_*p*_^*2*^ = .07), and mandatory waiting periods, (Experiment 1: *F*[1, 192] = 12.24, *p* = .001, *η*_*p*_^*2*^ = .06; Experiment 2: *F*[1, 693] = 38.12, *p* < .001, *η*_*p*_^*2*^ = .05), such that participants were more willing to work with gun owners to promote the policies than with non-gun owners (see Fig 2).

**Fig 3:**
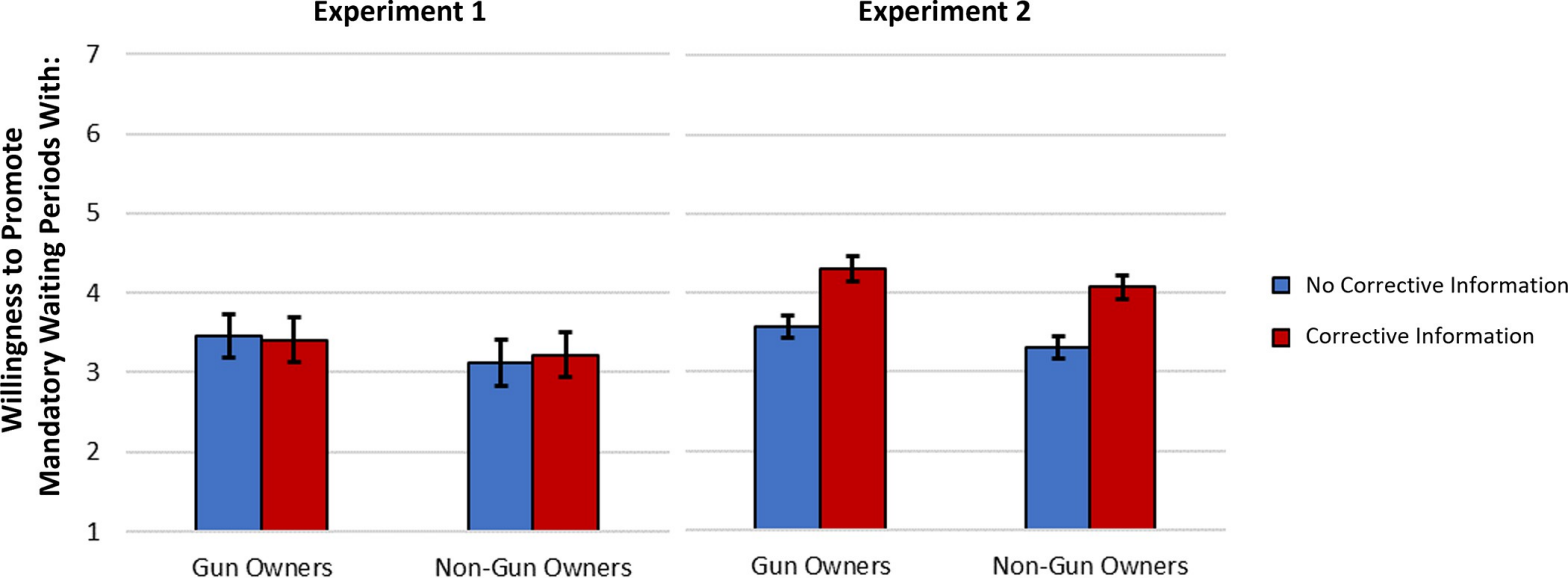
Willingness to promote mandatory waiting periods with gun and non-gun owners as a function corrective information condition. Data from Experiments 1 (*N* = 195 gun owners) and 2 (*N* = 696 gun owners). Error bars represent 95% confidence intervals.

Unexpectedly, in Experiment 1 the corrective information manipulation did not impact willingness to collaborate on universal background checks, *F*(1, 192) = 0.40, *p* = 0.53, *η*_*p*_^*2*^ = .002, or mandatory waiting periods, *F*(1, 192) = 0.008, *p* = .93, *η*_*p*_^*2*^ < .001. However, in Experiment 2 the manipulation did significantly impact willingness to collaborate on universal background checks, *F*(1, 693) = 15.36, *p* < .001, *η*_*p*_^*2*^ = .022, and mandatory waiting periods, *F*(1, 693) = 23.77, *p* < .001, *η*_*p*_^*2*^ = .033, such that those exposed to corrective information were more willing to collaborate (see Fig 3). The interaction between target and corrective manipulation was not significant in either experiment (*p*s > .19).

Because the main effect of the corrective information manipulation was significant in Experiment 2 but not Experiment 1, we tested whether this main effect would emerge when the data from both studies were combined. This is consistent with an integrative data analysis approach that allows for better power to detect an effect when all relevant data is considered compared to when studies are examined individually [32]. To test whether the main effect of the corrective information manipulation were significant across all of our data, we combined the data from Experiments 1 and 2 and tested two multilevel models: one predicting willingness to promote universal background checks and the other predicting willingness to promote mandatory waiting periods. These outcomes were predicted from the corrective information manipulation, target, and their interaction while controlling for participant gender. Experiment and participant were included as random factors. Significant effects of the corrective manipulation paralleling those in Experiment 2 emerged predicting willingness to collaborate on universal background checks, *b* = 0.20, *se* = .06, *t*(887.99) = 3.11, *p* = .002, 95% CI [.075, .327], and mandatory waiting periods, *b* = 0.28, *se* = .06, *t*(887.89) = 4.32, *p* < .001, 95% CI [.155, .410]. Therefore, receiving corrective information does appear to lead to greater willingness to work with others to promote gun safety policies, and this effect emerges robustly when all relevant data are considered.

#### Negative affect towards gun and non-gun owners

**Fig 4:**
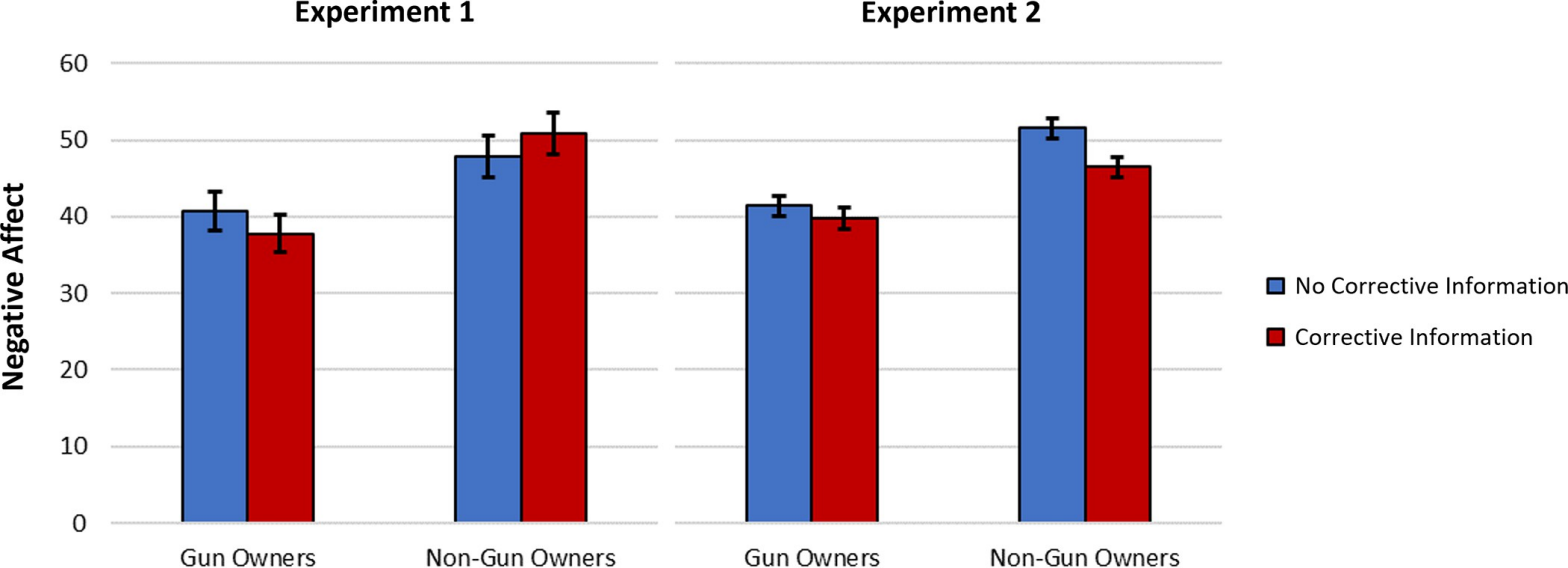
Negative affect towards gun and non-gun owners as a function of corrective information condition. Data from Experiments 1 (*N* = 195 gun owners) and 2 (*N* = 696 gun owners). Higher values indicate more negative affect. Error bars represent 95% confidence intervals.

Another repeated measures ANOVA was used to predict negative affect towards gun and non-gun owners. There was a main effect of rating target, such that greater negative affect was reported towards non-gun owners than gun owners, (Experiment 1: *F*[1, 192] = 24.08, *p* < .001, *η*_*p*_^*2*^ = .11; Experiment 2: *F*[1, 693] = 73.57, *p* < .001, *η*_*p*_^*2*^ = .10). As with the willingness to cooperate outcome measures, there was no main effect of the corrective information manipulation in Experiment 1, *F*(1, 192) = 0.001, *p* = .98, *η*_*p*_^*2*^ < .001, but there was a significant main effect in Experiment 2, *F*(1, 693) = 11.54, *p* = .001, *η*_*p*_^*2*^ = .02, such that those the corrective condition(s) reported less animosity towards the groups (see Fig 4). There was no significant interaction in either experiment (*p*s > .08).

We again conducted a multilevel model predicting negative affect across all of our data from Experiments 1 and 2. Paralleling Experiment 2, a significant main effect of the corrective information manipulation on negative affect towards gun and non-gun owners emerged, *b* = -1.24, *se* = .43, *t*(1776.00) = -2.86, *p* = .004, 95% CI [-2.080, -0.391]. This suggests that receiving corrective information does reduce negative affect towards outgroup members as well as ingroup members, and this effect emerges robustly when all relevant data are considered.

## Discussion

The widespread misperception among gun owners that most other gun owners oppose gun safety policies may help to explain why gun owners who support these policies do not more actively promote them. The resulting absence of public action by gun owners in support of such legislation has contributed significantly to the slow pace of policy change on this issue [17, 25]. Correcting misperceptions about gun owners’ attitude toward gun safety laws could be a critical tool for enhancing their attitudes toward, and their willingness to collaborate with, non-gun owners to enact gun safety laws.

Across two experiments, correcting gun owners’ misperceptions about support for gun safety policies among other gun owners increased perceptions of group identity overlap between gun and non-gun owners, supporting our first hypothesis. The effect sizes for this effect across experiments were similar to median effect sizes observed in social behavioral research [33], suggesting that these effects are meaningful. Experiment 2 found that correcting these misperceptions also increased willingness to work with both gun and non-gun owners to promote gun safety policies and reduced negative affect towards both groups. Effect sizes for these effects were small to medium based on commonly applied rules of thumb for evaluating partial eta squared effects sizes [34], though these effects were not observed in Experiment 1. Given the similarities between experiments, it is unclear why these effects differed between experiments. One possibility is the larger sample analyzed in Experiment 2 afforded better statistical power to detect the effects. Another possibility is the difference in when these studies were conducted temporally. Experiment 1 was conducted more closely following the January 6^th^ insurrection at the United States Capitol than Experiment 2. Given the salience of partisan division following that event, it is possible that participants in Experiment 1 were basing their willingness to cooperate judgments more on their partisan affiliations than on their gun owner identity and/or perceptions of fellow gun owner support for the policies. Regardless, because significant effects of the corrective information manipulation on these variables emerged across all data we collected, these effects appear robust overall. Therefore, Hypotheses 2 and 3 were supported. These effects did not appear to be dependent on the provision of an explanation for why gun owner support for gun safety policy is underestimated, so Hypothesis 4 was not supported.

The present results align with research examining social norms and intergroup dynamics. When participants’ perceptions of what was normative for ingroup members to believe were updated via exposure to corrective information, they became more willing to act in accordance with those beliefs. This suggests that normative misperceptions might undermine gun safety promotion even among people who support those policies. Additionally, correcting these misperceptions altered views of intergroup differences between gun and non-gun owners, with those exposed to the corrective information seeing non-gun owners as more similar to their group and expressing less animosity towards them.

It was interesting that the corrective information also increased willingness to cooperate with other gun owners to promote the policies and to reduced negative feelings towards them. This suggests that when gun owners misperceive other gun owners’ beliefs about gun safety policies, it undermines their willingness to work with anyone, be they gun owners or non-gun owners, to promote those policies. Additionally, gun owners could harbor animosity towards ingroup members they believe hold incorrect positions or who might be making their group look bad [35]. As such, it is possible that gun owners who support gun safety policies hold some resentment towards others in their group whom they mistakenly believe to oppose these policies. Learning that fewer ingroup members hold those positions than originally believed could then reduce negativity felt towards those ingroup members. As such, corrective information appears effective in facilitating both inter and intragroup cooperation to promote gun safety policies.

### Limitations

The present research had several limitations. First, this work relied on Mechanical Turk samples which have been shown to differ from nationally representative samples. Mechanical Turk samples have been found to be younger, less racially diverse, and more liberal than nationally representative samples [36]. Future research should seek to replicate the present findings using a more nationally representative sample to ensure the generalizability of the present findings to the broader United States population. Second, the present research focused on gun owners, so it is unclear whether corrective information would also impact non-gun owners. Future research should sample non-gun owners and test if corrective information increases their willingness to cooperate with gun-owners. Third, the sample sizes used for the individual studies reported in the present research might have been too small to offer ideal statistical power on their own, with a combination of the data offering a potentially better test of key predictions. Future research might benefit from recruiting larger sample sizes for each individual study.

Additionally, the present research did not take into account possible source effects that could enhance or undermine recipients’ acceptance of corrective information. Persuasion research finds that characteristics of the source of a message, such how trustworthy they are perceived to be, can impact that message’s persuasiveness [37]. Given the contentious debate around gun safety policies, it is possible that gun owners would mistrust corrective information from certain sources, such as vocal gun safety groups, who might be seen as opponents. Future research should test whether the source of corrective information might influence gun owners’ receptivity to that information. Finally, although willingness to engage in intergroup cooperation was enhanced by corrective information in the present research, whether this willingness would translate into actual cooperative behavior is unclear. Future research should incorporate behavioral measures, such as donations to non-gun owner-led gun safety organizations, to examine this.

### Conclusion

As Mark Twain noted, people often think they know “many things that just aren’t so.” This research found that gun owners think they “know” that other gun owners do not support gun safety policies, which just isn’t so—it is a misperception. Correcting gun owners’ misperceptions of gun owner support for gun safety policies appears to be an effective means to increase gun owners’ willingness to cooperate with non-gun owners to promote gun safety policies and to reduce outgroup animosity. Mobilization of gun owners in support of these policies might promote their adoption, which could help reduce gun violence in the United States [38].

## Supporting information

S1 File Supplemental information. This file contains additional details about the demographics of the reported studies’ samples, supplemental analyses, and specific study materials used in each study.
(DOCX)
