# Supplementary material for: Correcting misperceptions of gun policy support can foster intergroup cooperation between gun owners and non-gun owners
Source: PLoS One. 2022 Jun 8;17(6):e0268601. doi: 10.1371/journal.pone.0268601 (PMC9176832; doi:10.1371/journal.pone.0268601)
Supplement: S1 File — This file contains additional details about the demographics of the reported studies’ samples, supplemental analyses, and specific study materials used in each study. (DOCX) [file pone.0268601.s001.docx]

*Supplemental Materials*

*for*

Correcting misperceptions of gun policy support can foster intergroup cooperation between gun owners and non-gun owners

**Experiment 1 Additional Sample Demographics:**

**English as a First Language**

| Is English Your First Language | Percent of Sample |
| --- | --- |
| Yes | 100.0% |
| No | 0.0% |

**Racial Identity**

| Race | Percent of Sample |
| --- | --- |
| White | 81.5% |
| Black or African American | 9.2% |
| Hispanic | 4.1% |
| Asian | 2.6% |
| Native American | 0.0% |
| Mixed | 2.1% |
| Prefer not to say | 0.0% |
| Other | 0.5% |

**Income Bracket**

| Income Level | Percent of Sample |
| --- | --- |
| <$13,999 | 5.6% |
| $14,000 - $29,999 | 12.8% |
| $30,000 – $59,999 | 29.2% |
| $60,000 - $109,999 | 38.5% |
| $110,000 - $179,999 | 11.3% |
| >$180,000 | 2.1% |
| Prefer not to say | 0.5% |

**Education Level**

| Education Level | Percent of Sample |
| --- | --- |
| Some high school, no diploma | 0.0% |
| High school graduate, diploma or the equivalent (for example: GED) | 8.7% |
| Some college credit, no degree | 12.3% |
| Trade/technical/vocational training | 6.2% |
| Associate degree | 14.4% |
| Bachelor’s degree | 40.5% |
| Master’s degree | 15.9% |
| Professional degree | 0.0% |
| Doctorate degree | 2.1% |

**Political Partisan Affiliation**

| Affiliation | Percent of Sample |
| --- | --- |
| A Strong Democrat | 10.3% |
| A Not Very Strong Democrat | 7.2% |
| Independent, lean toward Democrat | 16.9% |
| Independent (close to neither party) | 15.4% |
| Independent, lean toward Republican | 13.3% |
| A Not Very Strong Republican | 14.9% |
| A Strong Republican | 20.0% |
| Other | 2.1% |

**Political Ideology** (7pt. scale: *1* *-* *Extremely Liberal* to *7 - Extremely Conservative*)

|  | *Mean* | *Standard Deviation* |
| --- | --- | --- |
| Ideology | 4.41 | 1.75 |

**Economic Conservatism** (7pt. scale: *1* – *I think Economic Equality is much more important* to *7 - I think Economic Freedom is much more important*)

|  | *Mean* | *Standard Deviation* |
| --- | --- | --- |
| Economic Conservatism | 4.54 | 2.02 |

**Social Conservatism** (7pt. scale: *1* – *I lean much more in the conservative direction* to *7 - I lean much more in the liberal direction*)

|  | *Mean* | *Standard Deviation* |
| --- | --- | --- |
| Ideology | 3.93 | 1.92 |

**Experiment 2 Additional Sample Demographics:**

**English as a First Language**

| Is English Your First Language | Percent of Sample |
| --- | --- |
| Yes | 99.1% |
| No | 0.9% |

**Racial Identity**

| Race | Percent of Sample |
| --- | --- |
| White | 83.8% |
| Black or African American | 5.5% |
| Hispanic | 4.9% |
| Asian | 2.6% |
| Native American | 0.7% |
| Mixed | 1.4% |
| Prefer not to say | 0.3% |
| Other | 0.9% |

**Income Bracket**

| Income Level | Percent of Sample |
| --- | --- |
| <$13,999 | 6.5% |
| $14,000 - $29,999 | 12.1% |
| $30,000 – $59,999 | 34.3% |
| $60,000 - $109,999 | 31.0% |
| $110,000 - $179,999 | 12.1% |
| >$180,000 | 2.2% |
| Prefer not to say | 1.9% |

**Education Level**

| Education Level | Percent of Sample |
| --- | --- |
| Some high school, no diploma | 0.9% |
| High school graduate, diploma or the equivalent (for example: GED) | 10.9% |
| Some college credit, no degree | 17.7% |
| Trade/technical/vocational training | 4.5% |
| Associate degree | 12.6% |
| Bachelor’s degree | 36.9% |
| Master’s degree | 13.6% |
| Professional degree | 1.7% |
| Doctorate degree | 1.1% |

**Political Partisan Affiliation**

| Affiliation | Percent of Sample |
| --- | --- |
| A Strong Democrat | 12.6% |
| A Not Very Strong Democrat | 11.1% |
| Independent, lean toward Democrat | 11.2% |
| Independent (close to neither party) | 15.1% |
| Independent, lean toward Republican | 11.6% |
| A Not Very Strong Republican | 15.4% |
| A Strong Republican | 20.8% |
| Other | 2.2% |

**Political Ideology** (7pt. scale: *1* *-* *Extremely Liberal* to *7 - Extremely Conservative*)

|  | *Mean* | *Standard Deviation* |
| --- | --- | --- |
| Ideology | 4.26 | 1.79 |

**Economic Conservatism** (7pt. scale: *1* – *I think Economic Equality is much more important* to *7 - I think Economic Freedom is much more important*)

|  | *Mean* | *Standard Deviation* |
| --- | --- | --- |
| Ideology | 4.56 | 1.96 |

**Social Conservatism** (7pt. scale: *1* – *I lean much more in the conservative direction* to *7 - I lean much more in the liberal direction*)

|  | *Mean* | *Standard Deviation* |
| --- | --- | --- |
| Social Conservatism | 3.95 | 1.96 |

**Analyses Comparing Estimates of Non-Gun Owner Support for Gun Safety Policies as a Function of the Corrective Information Manipulations Controlling for Participant Gender in Experiments 1 and 2:**

| **Table S1.**  *Estimates of Non-Gun Owner Support for Universal Background Checks Manipulation Check as a Function of Corrective Information Condition.* | | | | | | | | | | | |
| --- | --- | --- | --- | --- | --- | --- | --- | --- | --- | --- | --- |
|  |  | *Mean* | *SD* | *b* | *se* | *df* | *t* | *p* | *95% CI Lower* | *95% CI Upper* | *r* |
| Experiment 1 | No Corrective Information | 79.81 | 18.11 | 1.77 | 1.08 | 192 | 1.64 | .103 | -0.363 | 3.905 | .12 |
|  | Corrective Information | 83.36 | 11.28 |  |  |  |  |  |  |  |  |
| Experiment 2 | No Corrective Information | 81.62 | 15.24 | 0.45 | .58 | 693 | 0.78 | .437 | -.0.682 | 1.577 | .03 |
|  | Combined Corrective Information Conditions | 82.45 | 13.95 |  |  |  |  |  |  |  |  |

| **Table S2.**  *Estimates of Non-Gun Owner Support for Mandatory Waiting Periods Manipulation Check as a Function of Corrective Information Condition.* | | | | | | | | | | | |
| --- | --- | --- | --- | --- | --- | --- | --- | --- | --- | --- | --- |
|  |  | *Mean* | *SD* | *b* | *se* | *df* | *t* | *p* | *95% CI Lower* | *95% CI Upper* | *r* |
| Experiment 1 | No Corrective Information | 80.00 | 16.76 | 0.49 | 1.03 | 192 | 0.48 | .634 | -1.544 | 2.529 | .03 |
|  | Corrective Information | 80.99 | 11.57 |  |  |  |  |  |  |  |  |
| Experiment 2 | No Corrective Information | 79.97 | 16.09 | 0.62 | .58 | 693 | 1.07 | .285 | -0.514 | 1.745 | .04 |
|  | Combined Corrective Information Conditions | 81.20 | 13.43 |  |  |  |  |  |  |  |  |

**Analyses Comparing Effects of the Corrective Information Versus Corrective Plus Explanatory Information Conditions in Experiment 2:**

Note: These two conditions were combined for our main analyses because they did not produce different effects relative to each other, as can be seen in the analyses below.

| **Table S3.**  *Estimates of Gun Owner Support for Universal Background Checks and Mandatory Waiting Periods Manipulation Check as a Function of the Corrective and Corrective Plus Explanatory Conditions in Experiment 2.* | | | | | | | | | | | |
| --- | --- | --- | --- | --- | --- | --- | --- | --- | --- | --- | --- |
|  |  | *Mean* | *SD* | *b* | *se* | *df* | *t* | *p* | *95% CI Lower* | *95% CI Upper* | *r* |
| Universal Background Checks | Corrective Information | 77.25 | 15.96 | .53 | .74 | 452 | 0.71 | .476 | -0.924 | 1.977 | .03 |
|  | Corrective Plus Explanatory Information | 78.36 | 15.35 |  |  |  |  |  |  |  |  |
| Mandatory Waiting Periods | Corrective Information | 71.45 | 17.25 | .55 | .79 | 451 | 0.70 | .485 | -1.000 | 2.101 | .03 |
|  | Corrective Plus Explanatory Information | 72.64 | 16.20 |  |  |  |  |  |  |  |  |

| **Table S4.**  *Estimates of Identity Overlap Between Gun and Non-Gun Owners as a Function of the Corrective and Corrective Plus Explanatory Conditions in Experiment 2 Controlling for Gender.* | | | | | | | | | | |
| --- | --- | --- | --- | --- | --- | --- | --- | --- | --- | --- |
|  | *Mean* | *SD* | *b* | *se* | *df* | *t* | *p* | *95% CI Lower* | *95% CI Upper* | *r* |
| Corrective Information | 3.96 | 1.54 | -0.05 | .07 | 453 | -0.66 | .511 | -0.187 | 0.093 | .03 |
| Corrective Plus Explanatory Information | 3.84 | 1.49 |  |  |  |  |  |  |  |  |

| **Table S5.** | | | | |
| --- | --- | --- | --- | --- |
| *Willingness to Promote Universal Background Checks as a Function of the Corrective and Corrective Plus Explanatory Conditions in Experiment 2.* | | | | |
| Effect | *df* | *F* | *p* | *η_p_^2^* |
| Corrective Manipulation (CM; Corrective information vs. Corrective Explanatory Information) | 1, 453 | 2.43 | .120 | .005 |
| Target (Gun vs. Non-Gun Owners) | 1, 453 | 32.17 | < .001 | 0.07 |
| Gender | 1, 453 | 9.48 | .002 | .02 |
| CM*Target | 1, 453 | 0.05 | .825 | < .001 |
| Gender*Target | 1, 453 | 0.12 | .729 | < .001 |

| **Table S6.** | | | | |
| --- | --- | --- | --- | --- |
| *Willingness to Promote Mandatory Waiting Periods as a Function of the Corrective and Corrective Plus Explanatory Conditions in Experiment 2.* | | | | |
| Effect | *df* | *F* | *p* | *η_p_^2^* |
| Corrective Manipulation (CM; Corrective information vs. Corrective Plus Explanatory Information) | 1, 453 | 1.57 | .211 | .003 |
| Target (Gun vs. Non-Gun Owners) | 1, 453 | 27.92 | < .001 | 0.06 |
| Gender | 1, 453 | 4.95 | .027 | .011 |
| CM*Target | 1, 453 | 0.19 | .660 | < .001 |
| Gender*Target | 1, 453 | 0.41 | .522 | .001 |

| **Table S7.** | | | | |
| --- | --- | --- | --- | --- |
| *Negative Affect Towards Gun and Non-Gun Owners as a Function of the Corrective and Corrective Plus Explanatory Conditions in Experiment 2.* | | | | |
| Effect | *df* | *F* | *p* | *η_p_^2^* |
| Corrective Manipulation (CM; Corrective information vs. Corrective Plus Explanatory Information) | 1, 453 | 0.003 | .958 | < .001 |
| Target (Gun vs. Non-Gun Owners) | 1, 453 | 36.97 | < .001 | 0.08 |
| Gender | 1, 453 | 0.23 | .635 | < .001 |
| CM*Target | 1, 453 | 0.06 | .803 | < .001 |
| Gender*Target | 1, 453 | 0.02 | .899 | < .001 |

**Experimental Materials**

**Experiment 1:**

**First instructions:**

“Before we begin, we would like to ask you a couple questions about yourself.”

**Age measure:**

- What is your age? (please use only numbers in your answer) (open-ended response)

**Gun ownership measure**

- Do you own one or more firearms? (yes/no)

**Education**

- What is the highest degree or level of school you have completed? (If currently enrolled, highest degree received)
  - No schooling completed
  - Nursery school to 8th grade
  - Some high school, no diploma
  - High school graduate, diploma or the equivalent (for example: GED)
  - Some college credit, no degree
  - Trade/technical/vocational training
  - Associate degree
  - Bachelor’s degree
  - Master’s degree
  - Professional degree
  - Doctorate degree

**Second instructions:**

“We would now like to ask you a couple questions about your opinions on several of topics.”

**Gun regulation attitudes**

- Governmental policies to regulate gun ownership are: (-3 – Unfavorable to 3 – Favorable)
- How bad or good is gun control? (-3 – Bad to 3 – Good)
- To what extent are efforts by the government to regulate gun ownership negative or positive? (-3 – Negative to 3 – Positive)

**Universal background checks attitudes**

- Universal background checks for firearm purchases are: (-3 – Unfavorable to 3 – Favorable)
- How bad or good are universal background checks for firearm purchases? (-3 – Bad to 3 – Good)
- To what extent are universal background checks for firearm purchases negative or positive? (-3 – Negative to 3 – Positive)

**Mandatory waiting periods attitudes**

- Federal mandatory waiting periods for firearm purchases are:
- How bad or good are federal mandatory waiting periods for firearm purchases?
- To what extent are federal mandatory waiting periods for firearm purchases negative or positive?

**Sharing frequency**

- How often do you share your views on gun policy issues in-person with other people? (1 – Never to 7 – Very Often)
- How often do you share your views about gun policy issues with others online? (1 – Never to 7 – Very Often)

**Social identity measure (all items: -3 – Strongly Disagree to 3 – Strongly Agree)**

- When someone criticizes gun owners, it feels like a personal insult.
- I don’t act like the typical gun owner.
- I’m very interested in what others think about gun owners.
- The limitations associated with gun owners apply to me also.
- When I talk about gun owners, I usually say ‘‘we’’ rather than ‘‘they.’’
- I have a number of qualities typical of gun owners.
- Gun owners’ successes are my successes.
- If a story in the media criticized gun owners, I would feel embarrassed.
- When someone praises gun owners, it feels like a personal compliment.
- I act like a gun owner to a great extent.

**Identity importance**

- How important to you is your identity of being a gun owner? (1 – Not at All Important to 7 – Very Important)
- To what extent is being a gun owner central to your identity? (1 – Not at All to 7 – Very Much)

**Third instructions:**

“We are interested in how people think about and respond to issues surrounding gun ownership. Therefore, in this study we will ask you several questions about your thoughts and opinions about this issue.”

**Corrective information manipulation:**

*Corrective information condition*

“Before we ask you several questions, we would like to provide some additional context about support in the United States for several proposed gun safety policies. Specifically, we would like to provide context for **how popular these policies are among** **American gun owners.**

Recent national polling finds that a **sizable** **majority of American gun owners** support legislative action to curb gun violence. For instance, this polling finds that **85%** of **gun owners** support requiring universal background checks for all gun sales and **77%** support federal mandatory waiting periods for all gun purchases.

For comparison, recent polling finds that **87%** of **non-gun owners** support universal background checks and **84%** support mandatory waiting periods.

*No corrective information condition*

“Before we ask you several questions, we would like to provide some additional context about support in the United States for several proposed gun safety policies. Specifically, we would like to provide context for **how popular these policies are among** **Americans who do not own firearms.**

Recent national polling finds that **a sizeable majority of Americans non-gun owners** support legislative action to curb gun violence. For instance, this polling finds that **87%** of **non-gun owners** support requiring universal background checks for all gun sales and **84%** support federal mandatory waiting periods for all gun ownership purchases.”

**Fourth instructions:**

“We would now like to ask you several questions.”

**Perceptions of overlap measure**

Please select the number corresponding to the image that you think best represents the degree to which American gun owners and non-gun owners differ or do not differ from one another. Option 1 indicates that the two groups are totally different, option 7 indicates that there is virtually no difference between the two groups.


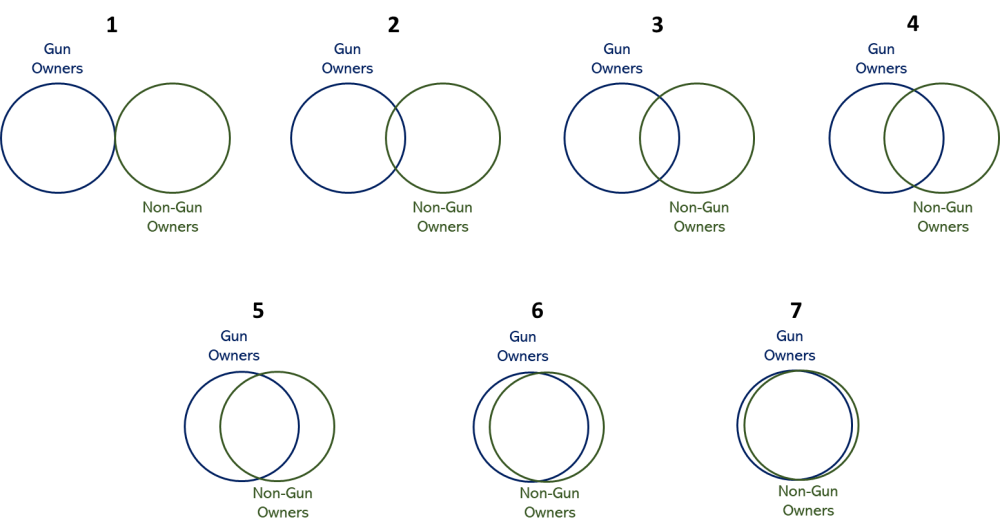


**Manipulation check items**

To the best of your knowledge:

- What percentage of American gun owners support requiring universal background checks for all gun sales? (Please only use numbers in your answer)
- What percentage of American gun owners support federal mandatory waiting periods for all gun purchases? (Please only use numbers in your answer)
- What percentage of American non-gun owners support requiring universal background checks for all gun sales? (Please only use numbers in your answer)
- What percentage of American non-gun owners support federal mandatory waiting periods for all gun purchases? (Please only use numbers in your answer)

**Desire for unity**

- To what extent do you want gun and non-gun owners to put their differences aside and come together? (1 – Not at All to 7 – Very Much)
- To what degree do you want unity between gun and non-gun owners? (1 – Not at All to 7 – Very Much)

**Willingness to dialogue with gun owners**

- How willing would you be to discuss gun regulation policies with someone who owns a gun? (1 – Not at All Willing to 7 – Extremely Willing)
- How open would you be to a discussion about gun law reform with someone who owns a firearm? (1 – Not at All Open to 7 – Extremely Open)

**Willingness to dialogue with non-gun owners**

- How willing would you be to discuss gun regulation policies with someone who does not own a gun? (1 – Not at All Willing to 7 – Extremely Willing)
- How open would you be to a discussion about gun law reform with someone who does not own a firearm? (1 – Not at All Open to 7 – Extremely Open)

**Willingness to associate with gun owners**

- How willing would you be to befriend someone who owns a firearm? (1 – Not at All Willing to 7 – Extremely Willing)
- How interested would you be in getting to better know people who own guns? (1 – Not at All Interested to 7 – Extremely Interested)

**Willingness to associate with non-gun owners**

- How willing would you be to befriend someone who does not own a firearm? (1 – Not at All Willing to 7 – Extremely Willing)
- How interested would you be in getting to better know people who do not own guns? (1 – Not at All Interested to 7 – Extremely Interested)

**Willingness to work with gun owners mandatory waiting periods**

- How willing would you be to work with gun owners to advocate for mandatory waiting periods for firearm purchases? (1 – Not at All Willing to 7 – Extremely Willing)
- How likely would you be to protest with gun owners in support of mandatory waiting periods for firearm purchases? (1 – Not at All Likely to 7 – Extremely Likely)
- How willing would you be to give money to an organization led by gun owners that advocates for mandatory waiting periods for firearm purchases? (1 – Not at All Willing to 7 – Extremely Willing)

**Willingness to work with non-gun owners mandatory waiting periods**

- How willing would you be to work with non-gun owners to advocate for mandatory waiting periods for firearm purchases? (1 – Not at All Willing to 7 – Extremely Willing)
- How likely would you be to protest with non-gun owners in support of mandatory waiting periods for firearm purchases? (1 – Not at All Likely to 7 – Extremely Likely)
- How willing would you be to give money to an organization led by non-gun owners that advocates for mandatory waiting periods for firearm purchases? (1 – Not at All Willing to 7 – Extremely Willing)

**Willingness to work with gun owners universal background checks**

- How willing would you be to work with gun owners to advocate for universal background checks for firearm purchases? (1 – Not at All Willing to 7 – Extremely Willing)
- How likely would you be to protest with gun owners in support of universal background checks for firearm purchases? (1 – Not at All Likely to 7 – Extremely Likely)
- How willing would you be to give money to an organization led by gun owners that advocates for universal background checks for firearm purchases? (1 – Not at All Willing to 7 – Extremely Willing)

**Willingness to work with non-gun owners universal background checks**

- How willing would you be to work with non-gun owners to advocate for universal background checks for firearm purchases? (1 – Not at All Willing to 7 – Extremely Willing)
- How likely would you be to protest with non-gun owners in support of universal background checks for firearm purchases? (1 – Not at All Likely to 7 – Extremely Likely)
- How willing would you be to give money to an organization led by non-gun owners that advocates for universal background checks for firearm purchases? (1 – Not at All Willing to 7 – Extremely Willing)

**Negative affect towards gun owners**

To what extent do you disagree or agree with the following:

Gun owners are: (100pt. sliders: -50 – Strongly Disagree to 50 – Strongly Agree)

- Patriotic
- Close-minded
- Intelligent
- Hypocritical
- Selfish
- Honest
- Open-minded
- Generous
- Mean

**Negative affect towards non-gun owners**

To what extent do you disagree or agree with the following:

Gun owners are: (100pt. sliders: -50 – Strongly Disagree to 50 – Strongly Agree)

- (same adjectives as in the negative affect towards gun owners measure)

**Fifth instructions**

“We would now like to ask you several additional questions about yourself:”

**Gender**

- Male
- Female
- Non-binary
- Prefer not to say
- Other (open-ended)

**Race**

- White
- Black or African American
- Hispanic
- Asian
- Native American
- Mixed
- Prefer not to say
- Other (open-ended)

**ESL**

- Is English your first language? (yes/no/unsure)

**Income**

- Which annual income bracket do you fall into?
  - <$13,999
  - $14,000 - $29,999
  - $30,000 - $59,999
  - $60,000 - $109,999
  - $110,000 - $179,999
  - >$180,000
  - Prefer not to say

**Political ideology**

- Now when thinking about politics, how would you describe your political views?
  - Very Liberal
  - Liberal
  - Somewhat Liberal
  - Moderate or Middle of the Road
  - Somewhat Conservative
  - Conservative
  - Very Conservative

**Political partisan affiliation**

- A Strong Democrat
- A Not Very Strong Democrat
- Independent, lean toward Democrat
- Independent (close to neither party)
- Independent, lean toward Republican
- A Not Very Strong Republican
- A Strong Republican
- Other, please specify (open-ended response)

**Economic conservatism**

- Some people believe that it is most important to reduce the economic differences in society even if it leads to a redistribution of resources from those who have a lot to those who have little. Others believe that each individual has the right to reap the results of his or her financial success even if it leads to economic inequality in society. Using the scale shown below, how would you describe your beliefs? (1 - I think Economic Equality is much more important to 7 - I think Economic Freedom is much more important)

**Social conservatism**

- People who are socially conservative emphasize the value of societal traditions and of sometimes getting into line and following norms. People who are socially liberal emphasize the value of individual freedom and the right of all individuals to fully be themselves. Using the scale shown below, how would you describe your beliefs? (1 - I lean much more in the Conservative direction to 7 - I lean much more in the Liberal direction)

**Comment**

“Do you have any comments you'd like to share about this study?”

**Hypothesis**

What do you think is the hypothesis being tested in this study?

**Winograd questions (open-ended responses)**

- Please answer the following question: Bob, Sally, and Jane all have apples. Sally and Jane give away their apples. Who still has an apple?
- Please answer the following question: Jackie, Harry, and Sam are standing in a line to buy cookies. Jackie is first, followed by Harry, followed by Sam. If Jackie moves to the back of the line, who is now first in line?

**Experiment 2:**

**First instructions:**

Same as Experiment 1.

**Age measure:**

Same as Experiment 1.

**Gun ownership measure**

Same as Experiment 1.

**Education**

Same as Experiment 1.

**Second instructions:**

Same as Experiment 1.

**Gun regulation attitudes**

Same as Experiment 1.

**Universal background checks attitudes**

Same as Experiment 1.

**Mandatory waiting periods attitudes**

Same as Experiment 1.

**Social identity measure (all items: -3 – Strongly Disagree to 3 – Strongly Agree)**

Same as Experiment 1.

**Third instructions:**

Same as Experiment 1.

**Corrective manipulations:**

*Corrective information condition*

“Before we ask you several questions, we would like to provide some additional context about support in the United States for several proposed gun safety policies. Specifically, we would like to provide context for **how popular these policies are among** **American gun owners.**

Recent national polling finds that a **sizable** **majority of American gun owners** support legislative action to curb gun violence. For instance, this polling finds that **85%** of **gun owners** support requiring universal background checks for all gun sales and **77%** support federal mandatory waiting periods for all gun purchases.

For comparison, recent polling finds that **87%** of **non-gun owners** support universal background checks and **84%** support mandatory waiting periods.

*Corrective plus opinion climate condition*

“Before we ask you several questions, we would like to provide some additional context about support in the United States for several proposed gun safety policies. Specifically, we would like to provide context for **how popular these policies are among** **American gun owners.**

Recent national polling finds that a **sizable** **majority of American gun owners** support legislative action to curb gun violence. For instance, this polling finds that **85%** of **gun owners** support requiring universal background checks for all gun sales and **77%** support federal mandatory waiting periods for all gun purchases.

For comparison, recent polling finds that **87%** of **non-gun owners** support universal background checks and **84%** support mandatory waiting periods.

Though there is sizeable support for these policies among **gun and non-gun owners**, these policies are still often treated as if they are controversial. A possible reason for this is that a vocal minority of gun owners opposed to these policies tend to dominate discussions about them. This likely leads people to think these policies are more contentious than they are in reality.”

*No corrective information condition*

“Before we ask you several questions, we would like to provide some additional context about support in the United States for several proposed gun safety policies. Specifically, we would like to provide context for **how popular these policies are among** **Americans who do not own firearms.**

Recent national polling finds that **a sizeable majority of Americans non-gun owners** support legislative action to curb gun violence. For instance, this polling finds that **87%** of **non-gun owners** support requiring universal background checks for all gun sales and **84%** support federal mandatory waiting periods for all gun ownership purchases.”

**Intergroup trust manipulation**

*Intergroup trust bolstered condition*

“It is important to note that the widespread support for these policies does not mean that the majority of Americans are against gun ownership. On the contrary, the right to own guns is widely supported, with recent polling finding that over two thirds of Americans, including a majority of Democrats and Republicans, support the right to keep and bear arms. Therefore, efforts to enact these policies are unlikely to lead to efforts to enact stricter gun safety policies in the future.”

*Control condition*

[no additional information was presented in this condition]

**Fourth instructions:**

“We would now like to ask you several questions.”

**Perceptions of overlap measure**

Same as Experiment 1.

**Manipulation check items**

Same as Experiment 1.

**Desire for unity**

Same as Experiment 1.

**Willingness to dialogue with gun owners**

Same as Experiment 1.

**Willingness to dialogue with non-gun owners**

Same as Experiment 1.

**Willingness to associate with gun owners**

Same as Experiment 1.

**Willingness to associate with non-gun owners**

Same as Experiment 1.

**Willingness to work with gun owners mandatory waiting periods**

- Same as Experiment 1.
- Same as Experiment 1.
- How willing would you be to support an organization led by gun owners that advocates for mandatory waiting periods for firearm purchases? (1 – Not at All Willing to 7 – Extremely Willing)

**Willingness to work with non-gun owners mandatory waiting periods**

- Same as Experiment 1.
- Same as Experiment 1.
- How willing would you be to support an organization led by non-gun owners that advocates for mandatory waiting periods for firearm purchases? (1 – Not at All Willing to 7 – Extremely Willing)

**Willingness to work with gun owners universal background checks**

- Same as Experiment 1.
- Same as Experiment 1.
- How willing would you be to support an organization led by gun owners that advocates for universal background checks for firearm purchases? (1 – Not at All Willing to 7 – Extremely Willing)

**Willingness to work with non-gun owners universal background checks**

- Same as Experiment 1.
- Same as Experiment 1.
- How willing would you be to support an organization led by non-gun owners that advocates for universal background checks for firearm purchases? (1 – Not at All Willing to 7 – Extremely Willing)

**Negative affect towards gun owners**

Same as Experiment 1.

**Negative affect towards non-gun owners**

Same as Experiment 1.

**Fifth instructions**

Same as Experiment 1.

**Gender**

Same as Experiment 1.

**Race**

Same as Experiment 1.

**ESL**

Same as Experiment 1.

**Income**

Same as Experiment 1.

**Political ideology**

Same as Experiment 1.

**Political partisan affiliation**

Same as Experiment 1.

**Economic conservatism**

Same as Experiment 1.

**Social conservatism**

Same as Experiment 1.

**Comment**

Same as Experiment 1.

**Hypothesis**

Same as Experiment 1.

**Winograd questions (open-ended responses)**

Same as Experiment 1.
